# Serotype Diversity and Antimicrobial Resistance Profile of *Salmonella enterica* Isolates From Freshwater Turtles Sold for Human Consumption in Wet Markets in Hong Kong

**DOI:** 10.3389/fvets.2022.912693

**Published:** 2022-07-22

**Authors:** Violaine Albane Colon, Kittitat Lugsomya, Hoi Kiu Lam, Lloyd Christian Wahl, Rebecca Sarah Victoria Parkes, Catherine Anne Cormack, Jule Anna Horlbog, Marc Stevens, Roger Stephan, Ioannis Magouras

**Affiliations:** ^1^Department of Infectious Diseases and Public Health, Jockey Club College of Veterinary Medicine and Life Sciences, City University of Hong Kong, Kowloon, Hong Kong SAR, China; ^2^Centre for Applied One Health Research and Policy Advice, Jockey Club College of Veterinary Medicine and Life Sciences, City University of Hong Kong, Kowloon, Hong Kong SAR, China; ^3^Department of Veterinary Clinical Sciences, Jockey Club College of Veterinary Medicine and Life Sciences, City University of Hong Kong, Kowloon, Hong Kong SAR, China; ^4^Centre for Animal Health and Welfare, Jockey Club College of Veterinary Medicine and Life Sciences, City University of Hong Kong, Kowloon, Hong Kong SAR, China; ^5^Jockey Club College of Veterinary Medicine and Life Sciences, City University of Hong Kong, Kowloon, Hong Kong SAR, China; ^6^Institute for Food Safety and Hygiene, Vetsuisse Faculty, University of Zurich, Zürich, Switzerland; ^7^Vetsuisse Faculty, National Reference Center for Enteropathogenic Bacteria and Listeria (NENT), Institute for Food Safety and Hygiene, University of Zurich, Zürich, Switzerland

**Keywords:** *Salmonella*, turtles, antimicrobial resistance, *cfr* gene, wet markets, zoonoses, Hong Kong

## Abstract

Chelonians are recognized as a source of human salmonellosis through direct contact or consumption of their meat. Freshwater turtles sold for food are widely available in wet markets in Asia. In this pilot study, 50 turtles belonging to three species were randomly sampled from wet markets throughout Hong Kong. The turtles were humanely euthanised and their feces or the colon were sampled for *Salmonella* culture. The *Salmonella* isolates obtained were serotyped and examined for phenotypic antimicrobial resistance and the presence of antimicrobial resistance genes. The study reports a high prevalence (42%, 95% CI: 29.4–55.8) and considerable serotype diversity of *Salmonella* among turtles sold in wet markets. The most common among the 11 serotypes isolated were *S*. Oranienburg and *S*. Thompson, which have been reported in turtles previously. The serotype *S*. Manhattan is reported in chelonians for the first time. Resistance to streptomycin and chloramphenicol was common, despite the latter being banned from aquaculture in mainland China since 2002. Resistance against fluoroquinolones and third-generation cephalosporins which represent first-line treatment options for salmonellosis was also observed. The multidrug-resistance gene *cfr* is identified for the first time in *Salmonella*. This is a worrying finding as it indicates an expansion of the *cfr* reservoir and potential horizontal spread to other bacteria. The results of this study emphasize the need for close surveillance of *Salmonella* from turtles sold as food and better regulation of turtle farming to safeguard public health and improve animal welfare.

## Introduction

Freshwater turtles are widely available in wet markets in South East Asia and Hong Kong and are primarily sold for consumption (1). A survey of 950,251 turtles for sale at wholesale and retail outlets in Hong Kong and Guangdong Province between 2000 and 2003 revealed that 77 different species, including endangered and critically endangered species, were sold (2). In Hong Kong, turtles are mostly imported from farms in Asian countries and mainland China, where large-scale turtle farming is estimated to be a multi-billion-dollar industry (1, 3). A study of 684 turtle farms in mainland China showed that ~127 million turtles across 11 different species are sold each year, of which the most common is the Chinese softshell turtle (SS) (*Pelodiscus sinensis*), accounting for over 97.6% of turtles sold (3).

There are numerous biological risks associated with the consumption of reptile products, including infections caused by bacteria, parasites, and exposure to biotoxins (4). *Salmonellae* are part of the normal intestinal flora of turtles, and turtle-associated salmonellosis has been a recognized public health issue for several decades (5, 6). While a high proportion of these infections is attributed to contact of young children with pet turtles (6, 7), salmonellosis has also been linked to the consumption of green turtles (*Chelonia mydas*) in Australia (8, 9) and snapping turtles (*Chelydra serpentina*) in Japan (10). Of the 2,659 *Salmonella (S.)* serotypes (11), seven have been implicated in reptile-associated salmonellosis in humans. These include *S*. Paratyphi B var Java, *S*. Poona, *S*. Pomona, *S*. Marina, *S*. Stanley, *S*. Litchfield, and *S*. Newport, and the most commonly reported *S*. Typhimurium and *S*. Enteritidis (6, 7).

*Salmonellae* are estimated to cause 93.8 million cases of gastroenteritis and 155,000 deaths globally each year (12). In Hong Kong, salmonellosis was the second most common bacterial cause of food poisoning from 2003 to 2011, with 3,250 cases (13). Although no cases of salmonellosis associated with turtle meat consumption in Hong Kong are published to date, close surveillance of *Salmonella* from all potential sources is essential to safeguard public health and for the timely detection of emerging serotypes. Furthermore, the ongoing spread of antimicrobial resistance (AMR) and the risk of dissemination of AMR genes (ARGs) in the population represents an additional challenge associated with *Salmonella* infections (14). Infections with resistant *Salmonella* are harder to treat and cause increased morbidity and mortality rates (15, 16).

There is limited information available on turtle farming practices, including antimicrobial usage, despite the large number of turtles farmed and consumed in Asia. Data on AMR in *Salmonella* isolated from turtles destined for human consumption is also sparse, as most studies have focused on wild turtles or captive turtles raised for the pet industry. One study from wet markets in Shanghai reported that most isolates (84%) were resistant to multiple antimicrobials (>3) (17). Other studies have looked at AMR in *Salmonella* from captive populations of freshwater turtles (18, 19). Red-eared sliders (RES) (*Trachemys scripta elegans*) sold in pet shops carried *Salmonella* with resistance against tetracycline, gentamycin, kanamycin, streptomycin, and sulfamethoxazole/trimethoprim (19).

To the author's knowledge, no previous studies have characterized *Salmonella* in turtles sold for human consumption in Hong Kong and research from other regions in Asia is scarce. The objectives of this pilot study were (1) to describe the frequency and serotypes of *Salmonella* in freshwater turtles sold in wet markets for human consumption, and (2) to characterize the AMR profile of the *Salmonella* isolates using phenotypic and molecular approaches. A better understanding of the zoonotic risks from turtle meat will provide a basis for improving consumer's and other stakeholder's awareness. Furthermore, it will stimulate discussions on developing clear guidelines on turtle farming and sale that could benefit animal welfare and safeguard public health.

## Materials and Methods

A list of wet markets in Hong Kong was made using publicly available information from the website of the Food and Environmental Hygiene Department (20). Due to the lack of information on live turtle availability and trade characteristics in wet markets, all 94 wet markets were visited twice weekly over a period of 3 months, and those selling live turtles were recorded. Three turtle species were available, namely SS, RES and Chinese striped neck turtles (CSN, *Mauremys sinensis*). From the final list of 28 wet markets where live turtles were available, 21 wet markets were randomly selected, and 50 freshwater turtles were sampled twice between January and March 2021. The wet markets sampled were distributed across all districts of Kowloon (9 wet markets from 5 districts) and Hong Kong island (6 wet markets from 4 districts) and 6 out of 9 districts in the New Territories (7 wet markets). The country of origin and whether the turtles were wild-caught or farmed was recorded.

A physical examination was performed on each turtle to record the general health condition and any obvious external lesions or abnormalities. Physical examinations were performed by a board-certified reptile veterinarian. Data including sex, weight and age group were collected. The turtles were anesthetized by intravenous injections of alfaxalone (Alfaxan®, Jurox Pty Limited, Rutherford, NSW 2320, Australia) at a dose of 10–20 mg/kg. Once anesthesia was confirmed, euthanasia was induced by an intravenous injection of pentobarbital (Dorminal 20%, Alfasan, 3449 JA Worden, The Netherlands) at a dose of 100 mg/kg, following the American Veterinary Medical Association guidelines (21). Fecal samples, if present, were collected during post-mortem examination and placed into sterile tubes with Amies agar gel transport swab (Thermo Fisher Scientific Australia Pty Ltd., Melbourne, Australia). If no feces were present, the colon area was swabbed, and the swabs were stored in a similar manner to the feces. The turtles were collected over 22 days and samples were processed the same day the turtles were bought.

### *Salmonella* Isolation and Identification

Each sample was placed in 10 ml buffered peptone water (BPW; Thermo Fisher Scientific Australia Pty Ltd., Melbourne, Australia) and incubated at 37°C for 18 h. Following incubation, 0.1 ml of cultured BPW was used to inoculate 10 ml Rappaport-Vassiliadis Soya Peptone broth (RVS; bioMérieux, Marcy-l'Étoile, France) at 41.5°C for 24 h. Cultured RVS was streaked on Xylose Lysine Deoxycholate agar (XLD; Thermo Fisher Scientific Australia Pty Ltd., Melbourne, Australia) and incubated at 37°C for 24 h (22). *Salmonella Typhimurium* (ATCC:14028™) was used as positive control. Putative *Salmonella* colonies (black color) were selected for species identification by Matrix-Assisted Laser Desorption/Ionization Time-Of-Flight (MALDI-TOF; Bruker, Massachusetts, US) mass spectrometry and analyzed by MALDI Biotyper® (Bruker, Massachusetts, US).

### *Salmonella* Serotyping

The obtained *Salmonella* strains were initially serotyped at the National Center for Enteropathogenic Bacteria and Listeria (NENT) at the University of Zurich, Switzerland. Typing was performed by slide agglutination with commercially available antisera (Sifin Diagnostics GmbH, Berlin, Germany) according to the Kauffmann-White-Le Minor scheme.

### Antimicrobial Susceptibility Testing

Antimicrobial susceptibility testing against 15 antimicrobials was performed by Kirby-Bauer disk diffusion test. The antimicrobials included ampicillin; AMP (10 μg), cefotaxime; CTX (30 μg), ceftazidime; CAZ (30 μg), meropenem; MEM (10 μg), imipenem; IPM (10 μg), ertapenem; ETP (10 μg), ciprofloxacin; CIP (5 μg), streptomycin; S (10 μg), gentamicin; GEN (10 μg), amikacin; AMK (30 μg), sulfamethoxazole-trimethoprim; SXT (23.75 μg/1.25 μg), doripenem; DOR (10 μg), chloramphenicol; CHL (30 μg), azithromycin; AZM (15 μg) (Thermo Fisher Scientific Australia Pty Ltd., Melbourne, Australia). A colistin (CST) (0.5-32 μg/ml) (MilliporeSigma, Massachusetts, US) susceptibility test was performed using the broth microdilution test. The zone of inhibition and minimal inhibitory concentration were interpreted using the clinical breakpoints published by the Clinical and Laboratory Standards Institute (CLSI), M100 31st edition (23). Extended-Spectrum β-lactamases confirmatory test was performed by Combination Disk Test (23). Linezolid (LZD) and chloramphenicol (CHL) MIC testing for the isolated, *cfr* carrying *S*. IIIb 50:k:z was performed using Etest® strips (bioMérieux, Marcy-l'Étoile, France).

### Whole Genome Sequencing

Whole genome sequencing was used for final confirmation and was performed as described previously (24). Briefly, paired-end libraries were produced and sequenced on an Illumina MiniSeq sequencer (Illlumina, San Diego, CA, USA). Reads were assembled using Spades 3.13.1 (25) in Shovill 1.0.4 (https://github.com/tseemann/shovill). Whole genome-based *Salmonella* Serotyping was performed using Seqsero (26) with standard settings. Antimicrobial resistance gene analysis was done using the NCBI AMRFinderPlus database (27) in Ridom Seqsphere v7.7.5 (Ridom GmbH, Münster, Germany) using standard settings.

### Statistical Analysis

Collection of datapoints and descriptive statistics were performed on an electronic spreadsheet (Excel, Microsoft Corp, Redmond, Wash). Association between pairs of variables was assessed by Fisher's exact test of independence using the online statistical tool Stangroom (28). Confidence intervals were calculated using the Wilson score method using the online calculator Epitools (29).

## Results

A total of 50 turtles were randomly sampled from 21 wet markets. The turtle species distribution was 16% (8/50) CSN, 22% (11/50) RES and 62% (31/50) SS (Table 1). Thirty-one turtles were male (62%) and all belonged to the species SS, and 19 turtles were female (38%) and were either CSN or RES. According to the information provided by the vendor, 30% (15/50) of turtles were wild-caught and 70% (35/50) were farmed. Thirteen of the wild turtles were SS and 2 were CSN. All farmed turtles originated from mainland China except for two turtles imported from Thailand. Four turtles were classified as juvenile (one CSN and three RES), while the rest were adult individuals. The mean weight of the turtles was 0.94 kg (median 0.92 kg, range 0.61–1.64 kg). The mean weight for RES was 0.84 kg, for SS it was 0.99 kg and for CSN was 0.81 kg. Feces was present in 68% (34/50) of the turtles (four CSN, 10 RES and 20 SS). The remaining animals (16/50) were sampled using swabs.

**Table 1:** The total number and proportion of turtles positive for *Salmonella enterica* by species, origin and sex.

**Species**	***N***	**% positive samples (n)**	**95% CI**
CSN	8	87.5 (7)	52.9–97.8
RES	11	72.7 (8)	43.4–90.3
SS	31	19.4 (6)	9.2–36.3
**Origin**
Farmed	35	51.4 (18)	35.6–67.0
Wild	15	20.0 (3)	7.0–45.2
**Sex**
Female	19	78.9 (15)	56.7–91.5
Male	31	9.4 (6)	9.2–36.3
**Total**	50	42.0 (21)	29.4–55.8

*CSN, Chinese stripe-necked turtle (Mauremys sinensis); RES, Red-eared slider (Trachemys scripta elegans); SS, Chinese softshell turtle (Pelodiscus sinensis)*.

**Figure 1:**
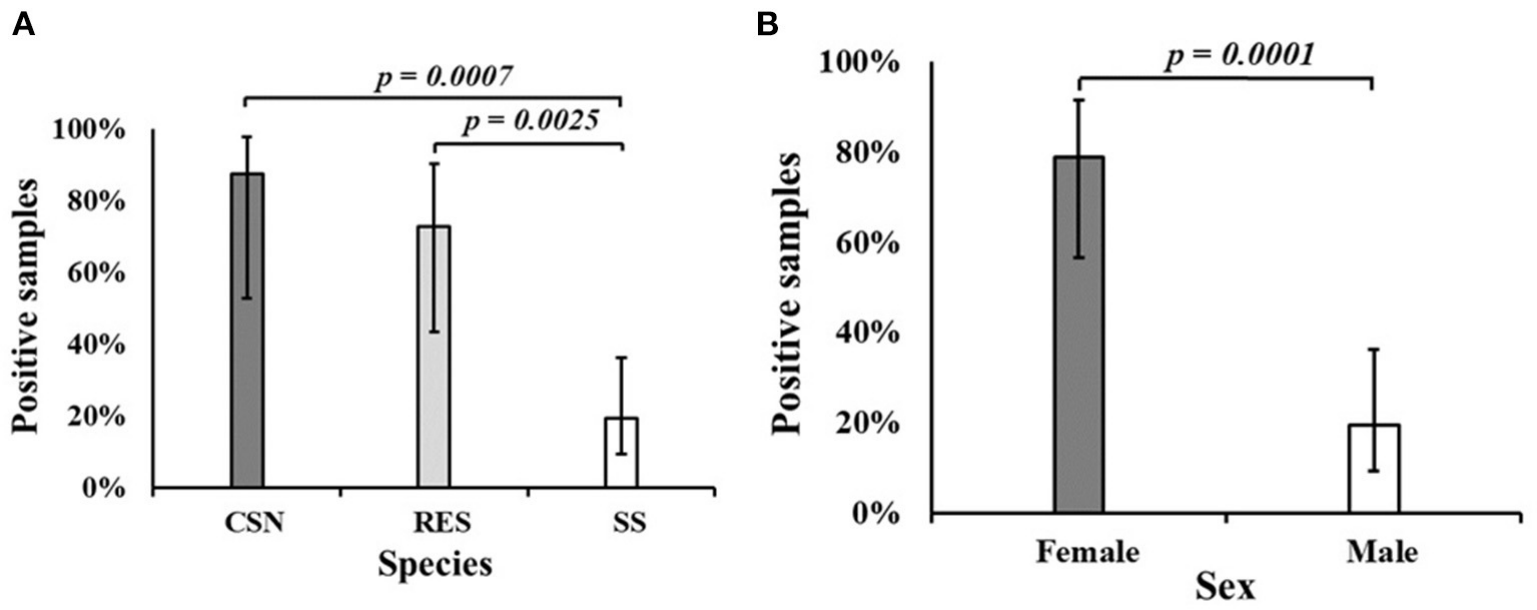
**(A)** The proportion of samples positive for *Salmonella enterica* by turtle species. **(B)** The proportion of samples positive for *Salmonella enterica* by sex. CSN, Chinese stripe-necked turtle (*Mauremys sinensis*); RES, Red-eared slider (*Trachemys scripta elegans*); SS, Chinese softshell turtle (*Pelodiscus sinensis*).

*Salmonella enterica* was isolated from 21 turtles (42%), including 87.5% of CSN (7/8), 72.7% of RES (8/11) and 19.4% of SS (6/31). A statistically significant difference (*p* < 0.001) was found between the proportion of positive SS and both CSN and RES (Figure 1A). A significant difference (*p* < 0.001) was found between the proportion of positive male (19.4%) and positive female turtles (78.9%) (Figure 1B). Feces were present in 34 turtles and 50.0% of these were positive for *S. enterica* (17/34) while 25.0% of turtles without feces were positive for *S. enterica* (4/16) although no significant difference was found (*p* = 0.129). A greater proportion of farmed turtles (50.4%) were positive for *S. enterica* compared to wild-caught (20%) samples although no significant difference was found (*p* = 0.061).

**Figure 2:**
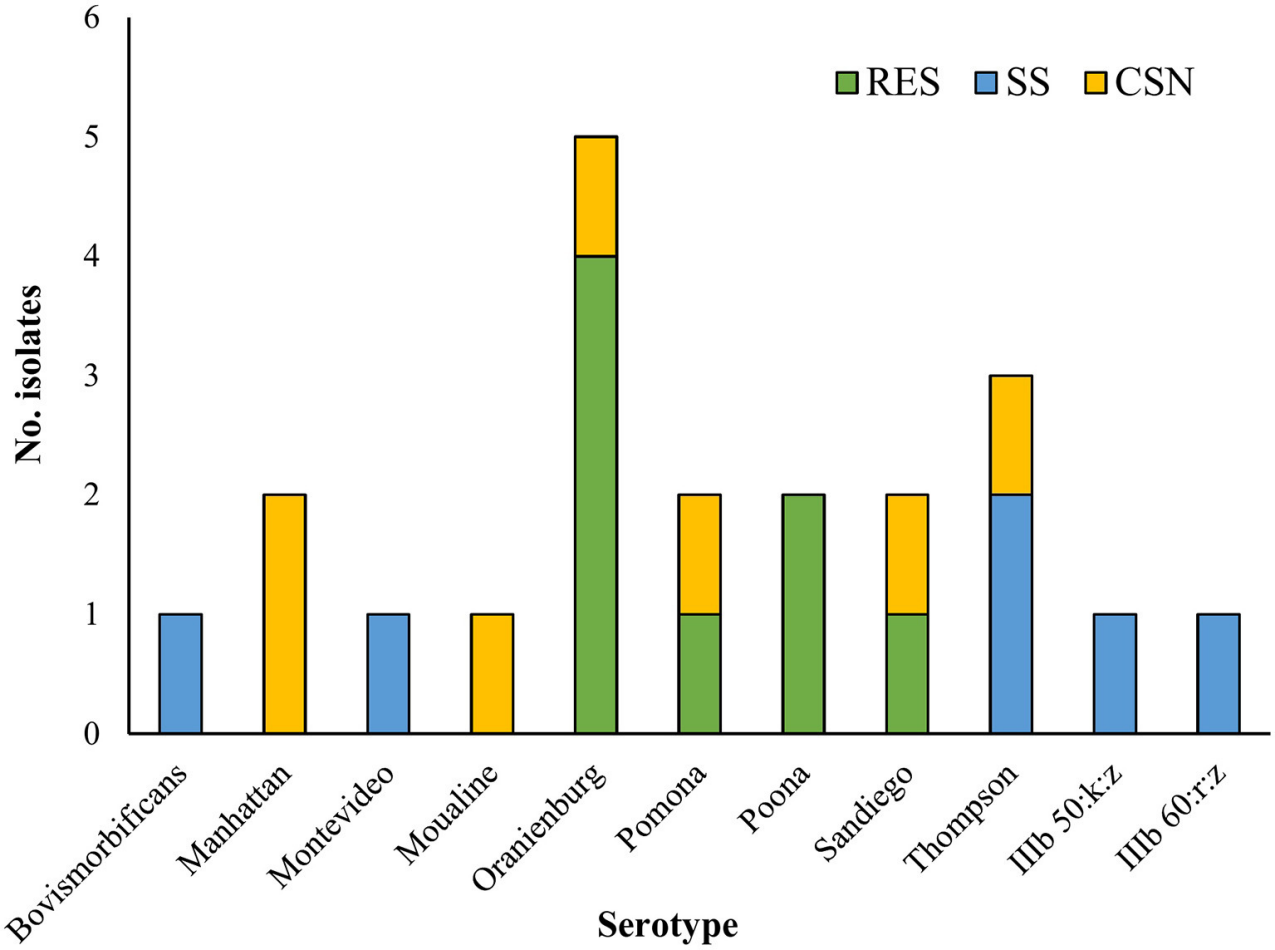
The number of *Salmonella enterica* isolates of each serotype isolated from different turtle species. CSN, Chinese stripe-necked turtle (*Mauremys sinensis*); RES, Red-eared slider (*Trachemys scripta elegans*); SS, Chinese softshell turtle (*Pelodiscus sinensis*).

Two isolates belonged to *S. enterica* subsp. *diarizonae* and the remaining nineteen isolates belonged to *S. enterica* subsp. *enterica*. The serotypes *S*. Oranienburg and *S*. Thompson were isolated 5 and 3 times respectively. There was no overlap between the serotype profile of isolates from RES, which included *S*. Oranienburg, *S*. Poona, *S*. Pomona and *S*. Sandiego, and the serotype profile of isolates from SS which included *S*. Bovismorbificans, *S*. Montevideo, *S*. Thompson, *S*. IIIb 50:k:z and *S*. IIIb 60:r:z (Figure 2). A list of *the S. enterica* isolates, including GenBank accession Numbers can be found in Table 2. All but one *S*. Oranienburg isolates were either sensitive or intermediate resistant to the antimicrobials tested. One isolate showed phenotypic resistance to azithromycin (Table 3). All three *S*. Thompson isolates were multidrug-resistant (i.e., resistant to at least one antimicrobial agent in three or more antimicrobial classes). In total, eight isolates (38.1%) were resistant to at least one antimicrobial, and four isolates (19%) were resistant to seven or more antimicrobials. Resistance to chloramphenicol (33.3%, 7/21) and streptomycin (28.6%, 6/21) were the most common phenotypes. Phenotypic resistance to the macrolide azithromycin was observed in five isolates. Phenotypic ciprofloxacin resistance was detected in four isolates and intermediate resistance in further four isolates (three *S*. Thompson and one *S*. Montevideo). No isolates were found to be carbapenem-resistant although three isolates (14.3%), two *S*. Thompson and one *S*. IIIb 50:k:z, were deemed to be ESBL-producing. A summary of the AMR phenotypes observed using disk diffusion test is shown in Table 3. The MIC values for chloramphenicol and linezolid in *S*. IIIb 50:k:z were ≥256 μg/ml for both antimicrobials.

**Table 2:** Serotypes, turtle species of origin and GenBank accession numbers of *Salmonella enterica* isolates.

**ID**	***Salmonella enterica* subspecies**	**Serotype**	**Turtle species**	**Accession Number**
1	*S. enterica* subsp. *enterica*	Bovismorbificans	SS	JAKMZU000000000
2	*S. enterica* subsp. *diarizonae*	IIIb 50:k:z	SS	JAKMZY000000000
3	*S. enterica* subsp. *diarizonae*	IIIb 60:r:z	SS	JAKMZM000000000
4	*S. enterica* subsp. *enterica*	Manhattan	CSN	JAKMZR000000000
5	*S. enterica* subsp. *enterica*	Manhattan	CSN	JAKMZQ000000000
6	*S. enterica* subsp. *enterica*	Montevideo	SS	JAKMZJ000000000
7	*S. enterica* subsp. *enterica*	Moualine	CSN	JAKMZL000000000
8	*S. enterica* subsp. *enterica*	Oranienburg	CSN	JAKNAC000000000
9	*S. enterica* subsp. *enterica*	Oranienburg	RES	JAKNAB000000000
10	*S. enterica* subsp. *enterica*	Oranienburg	RES	JAKMZX000000000
11	*S. enterica* subsp. *enterica*	Oranienburg	RES	JAKMZV000000000
12	*S. enterica* subsp. *enterica*	Oranienburg	RES	JAKMZS000000000
13	*S. enterica* subsp. *enterica*	Pomona	CSN	JAKMZT000000000
14	*S. enterica* subsp. *enterica*	Pomona	RES	JAKMZP000000000
15	*S. enterica* subsp. *enterica*	Poona	RES	JAKMZW000000000
16	*S. enterica* subsp. *enterica*	Poona	RES	JAKMZN000000000
17	*S. enterica* subsp. *enterica*	Sandiego	CSN	JAKMZO000000000
18	*S. enterica* subsp. *enterica*	Sandiego	RES	JAKMZK000000000
19	*S. enterica* subsp. *enterica*	Thompson	CSN	JAKNAA000000000
20	*S. enterica* subsp. *enterica*	Thompson	SS	JAKMZZ000000000
21	*S. enterica* subsp. *enterica*	Thompson	SS	JAKMZI000000000

*CSN, Chinese stripe-necked turtle (Mauremys sinensis); RES, Red-eared slider (Trachemys scriptaelegans); SS, Chinese softshell turtle (Pelodiscus sinensis)*.

**Table 3:** Antimicrobial sensitivity of *Salmonella enterica* isolates against individual antimicrobials using disk diffusion test.

**ID**	***Salmonella* serotype**	**CTX**	**CAZ**	**MEM**	**IPM**	**ETP**	**DOR**	**AMP**	**CIP**	**GEN**	**S**	**AMK**	**SXT**	**AZM**	**CHL**	**CST**	**ESBL-PE**	**CRE**	**Antibiogram**
1	Bovismorbificans	S	S	S	S	S	S	S	I	S	S	S	S	S	S	S	–	–	–
2	IIIb 50:k:z	R	R	S	S	S	S	R	I	S	R	S	R	R	R	S	ESBL-PE	–	CTX-CAZ-AMP-S-SXT-AZM-CHL
3	IIIb 60:r:z	S	S	S	S	S	S	S	S	S	S	S	S	S	S	S	–	–	–
4	Manhattan	S	S	S	S	S	S	S	I	S	R	S	I	S	R	S	–	–	S-CHL
5	Manhattan	S	S	S	S	S	S	S	I	S	R	S	S	S	R	S	–	–	S-CHL
6	Montevideo	S	S	S	S	S	S	R	R	S	S	S	R	S	R	S	–	–	AMP-CIP-SXT-CHL
7	Moualine	S	S	S	S	S	S	S	S	S	S	S	S	S	S	S	–	–	–
8	Oranienburg	S	S	I	S	S	S	S	S	S	S	S	S	S	S	S	–	–	–
9	Oranienburg	I	S	I	S	S	S	S	S	S	S	S	S	S	S	S	–	–	–
10	Oranienburg	S	S	S	S	S	S	S	S	S	S	S	S	R	S	S	–	–	AZM
11	Oranienburg	S	S	S	S	S	S	S	S	S	S	S	S	S	S	S	–	–	–
12	Oranienburg	S	S	S	S	S	S	S	S	S	S	S	S	S	S	S	-	-	–
13	Pomona	S	S	S	S	S	S	S	S	S	S	S	S	S	S	S	-	-	–
14	Pomona	S	S	S	S	S	S	S	S	S	S	S	S	S	S	S	-	-	–
15	Poona	S	S	S	S	S	S	S	S	S	S	S	S	S	S	S	–	–	–
16	Poona	S	S	S	S	S	S	S	S	S	S	S	S	S	S	S	–	–	–
17	Sandiego	S	S	S	S	S	S	S	S	S	S	S	S	S	S	S	–	–	–
18	Sandiego	S	S	S	S	S	S	S	S	S	S	S	S	S	S	S	–	–	–
19	Thompson	R	R	S	S	S	S	R	R	S	R	S	R	R	R	S	ESBL-PE	–	CTX-CAZ-AMP-CIP-S-SXT-AZM-CHL
20	Thompson	R	R	S	S	S	S	R	R	S	R	S	R	R	R	S	ESBL-PE	–	CTX-CAZ-AMP-CIP-S-SXT-AZM-CHL
21	Thompson	I	I	S	S	S	S	R	R	R	R	S	R	R	R	S	–	–	AMP-CIP-GEN-S-SXT-AZM-CHL
	*n* (%) resistant	3	3	0	0	0	0	5	4	1	6	0	5	5	7	0			
		(14.3)	(14.3)	(0)	(0)	(0)	(0)	(23.8)	(19)	(4.8)	(28.6)	(0)	(23.8)	(23.8)	(33.3)	(0)			

*S, sensitive; I, intermediate; R, resistant; CTX, cefotaxime; CAZ, ceftazidime; MEM, meropenem; IPM, imipenem; ETP, ertapenem; DOR, doripenem; AMP, ampicillin; CIP, ciprofloxacin; GEN, gentamicin; S, streptomycin; AMK, amikacin; SXT, trimethoprim-sulfamethoxazole; AZM, azithromycin; CHL, chloramphenicol; CST, colistin; ESBL-PE, extended-spectrum beta-lactamase-producing Enterobacteriaceae*.

Whole genome sequencing of the isolates revealed 33 unique ARGs spanning 11 antimicrobial groups (Tables 4a,b). The chloramphenicol and florfenicol resistance gene *floR*, and the sulphonamide resistance gene *sul2* were the most common ARGs, both present in 33.3% (7/21) of isolates. The aminoglycoside-inactivating phosphotransferase genes *aph(6)-Id* and *aph(3”)-Ib* were the second most common, co-occurring in the same 28.6% (6/21) of isolates. Furthermore, β-lactamase (BLAs) genes were found in 23.8% (5/21) of isolates. The *bla*_TEM−_1 and *bla*_CMY−2_ ARGs were isolated in all *S*. Thompson (*n* = 3) isolates. No ARGs were found in isolates belonging to *S*. Oranienburg (*n* = 5), *S*. Pomona (*n* = 2), *S*. Poona (*n* = 2) and *S*. IIIb 60:r:z (*n* = 1) serotypes. Antimicrobial resistance genes specific to aminoglycosides, amphenicols, sulphonamides and tetracyclines were identified in seven isolates, consisting of all *S*. Thompson (*n* = 3), *S*. Manhattan (*n* = 2), *S*. Montevideo (*n* = 1) and *S*. IIIb 50:k:z (*n* = 1) serotypes. The azithromycin resistance genes *mph(A)* and *erm(B)* were detected in one (*S*. Thompson) and three (one *S*. IIIb 50:k:z and two *S*. Thompson) isolates, respectively. Five quinolone ARGs were detected in seven different isolates. These included plasmid-mediated resistance genes *qnrS1, qnrA1, aac(6*′*)-Ib-cr* and *qepA8 S*. Moualine (*n* = 1), *S*. Sandiego (*n* = 2) and *S*. Bovismorbificans (*n* = 1) isolates which each only had ARGs for one single antimicrobial group, while the single *S*. IIIb 50:k:z isolate had resistance genes specific for ten different antimicrobial groups.

**Table 4a:** Antimicrobial resistance genes identified in *Salmonella enterica* isolates from turtles.

**ID**	**Serotype**	**Aminoglycosides**					**Amphenicols**	**Beta-lactams**		**Fosfomycin**
		**AMK**	**GEN**	**HYG**	**KAN**	**S**	**FLR/CHL**		**Cephalosporins**	**FOF**
1	Bovismorbificans									
2	IIIb 50:k:z	*aac(6')-Ib-cr5*			*aph(3')-Ia*	*aadA16 / aph(3”)-Ib / aph(6)-Id*	*floR*		*bla*_CMY−2_	
3	IIIb 60:r:z									
4	Manhattan					*aph(3”)-Ib / aph(6)-Id*	*floR*			
5	Manhattan					*aph(3”)-Ib / aph(6)-Id*	*floR*			
6	Montevideo	*aac(6')-Ib-cr5*	*aac(3)-IV*	*aph(4)-Ia*		*aadA2*	*catB3 / floR*	*bla*_OXA−1_		
7	Moualine									*fosA7*
8	Oranienburg									
9	Oranienburg									
10	Oranienburg									
11	Oranienburg									
12	Oranienburg									
13	Pomona									
14	Pomona									
15	Poona									
16	Poona									
17	Sandiego									*fosA7*
18	Sandiego									*fosA7*
19	Thompson				*aph(3')-Ia*	*aph(3”)-Ib / aph(6)-Id*	*floR*	*bla*_TEM−1_	*bla*_CMY−2_	
20	Thompson					*aph(3”)-Ib / aph(6)-Id*	*floR*	*bla*_TEM−1_	*bla*_CMY−2_	
21	Thompson				*aph(3')-Ia*	*aph(3”)-Ib / aph(6)-Id*	*floR*	*bla*_TEM−1_	*bla*_CMY−2_	*bla*_CMY−2_

*AMK, Amikacin; GEN, Gentamicin; HYG, Hygromycin B; KAN, Kanamycin; S, Streptomycin; FLR, Florfenicol; CHL, Chloramphenicol; FOF, Fosfomycin*.

**Table 4b:** Antimicrobial resistance genes identified in *Salmonella enterica* isolates from turtles.

**ID**	**Serotype**	**Lincosamides/Streptogramins**	**Macrolides**	**Quinolones**	**Rifamycins**	**Sulphonamides**		**Tetracyclines**
			**AZM/ERY/TEL/TYL**				**TMP**	**TET**
1	Bovismorbificans			*gyrA_S83F*				
2	IIIb 50:k:z	*cfr*	*cfr* / *erm*(B) / *mph*(E) */ msr*(E)	*aac(6')-Ib-cr5*	*arr-3*	*sul1 / sul2*	*dfrA12 / dfrA27*	*tet*(A)
3	IIIb 60:r:z							
4	Manhattan			*qnrS1*		*sul2*		*tet*(D)
5	Manhattan			*qnrS1*		*sul2*		*tet*(D)
6	Montevideo			*aac(6')-Ib-cr5 / qnrA1*	*arr-3*	*sul1 / sul2*	*dfrA12*	*tet*(B)
7	Moualine							
8	Oranienburg							
9	Oranienburg							
10	Oranienburg							
11	Oranienburg							
12	Oranienburg							
13	Pomona							
14	Pomona							
15	Poona							
16	Poona							
17	Sandiego							
18	Sandiego							
19	Thompson		*erm*(B) / *mph*(E) / *msr*(E)	*qepA8 / qnrS1*	*arr-2*	*sul1 / sul2*	*dfrA12 / dfrA14*	*tet*(A)
20	Thompson		*mph*(A)			*sul2*		*tet*(A)
21	Thompson		*erm*(B) / *mph*(E) / *msr*(E)	*qepA8 / qnrS1*	*arr-2*	*sul1 / sul2*	*dfrA12 / dfrA14*	*tet*(A)

*AZM, Azithromycin; ERY, Erythromycin; TEL, Telithromycin; TYL, Tylosin; TMP, Trimethoprim; TET, Tetracycline*.

## Discussion

This study found a high proportion of *Salmonella* carriage with a variety of AMR phenotypes and genotypes in turtles sold for food in wet markets throughout Hong Kong. A higher proportion of turtles sampled randomly from wet markets in Hong Kong (42%, 21/50) were positive for *S. enterica* than in a previous study from Shanghai, China (29.7%, 51/172), in which only SS were sampled (17). In the current study, 19.4% of SS were positive for *Salmonella*, significantly (*p* < 0.001) fewer than CSN (87.5%) and RES (72.7%). Published data for *Salmonella* in chelonians varies across different studies and different turtle populations, therefore findings are difficult to compare between studies. In Japan, the prevalence of *S. enterica* among pet shop RES was 53.7% (130/227) (19). In Shanghai, prevalence among pet turtles (species not specified) was found to be 18.9% (31/164) (17) and in pet turtles (several species) from Korea it was 50% (17/34) (18). In a pet shop in Spain, the proportion of turtles positive for *S. enterica* was 75.0% (18/24) (30).

The most common *Salmonella* serotypes isolated from turtles in this study were Oranienburg and Thompson. The latter was—together with *S*. Typhimurium—one of the two predominant serotypes identified in native European pond turtle (*Emys orbicularis*) and introduced RES in natural ponds in Spain (31). In addition, *S*. Thompson was also the most common serotype (17%, 14/85) isolated from SS and pet turtles in wet markets in Shanghai, China (17). *Salmonella* Oranienburg and *S*. Sandiego were isolated from RES sold in pet shops in Japan (19). *Salmonella* Manhattan is the only serotype isolated from turtles in the current study that—to the best of the author's knowledge—has never been reported in chelonians before. *Salmonella* Manhattan has been isolated from other reptiles, including Iguanas (*Conolophus subcristatus*) from the Galápagos Islands (32), captive Andros and Bahamian rock iguanas (*Cyclura cychlura* and *Cychlura rileyi*) (33), and northern water snakes (*Nerodia sipedon sipedon*) (34). Some of the serotypes identified here have also been linked to human salmonellosis. *Salmonella* Pomona, which is considered to be particularly pathogenic (35), caused ~18% of human salmonellosis cases due to turtle exposure in the USA between 2006 and 2014 (5). This serotype is particularly prevalent in turtles and other reptiles, as it has been found in 39% of free-living introduced RES caught in mainland China (35), and 12% of reptiles sampled from pet shops in Spain (36). *Salmonella* Sandiego was identified in three and *S*. Pomona and *S*. Poona each in two out of eight outbreaks of turtle-associated salmonellosis in young children during 2011–2013 across 41 states of the USA (37). *Salmonella* Thompson, along with *S*. Typhimurium, was among the four most-frequently recovered serotypes from human patients in Shanghai (38). In Hong Kong, the five main serotypes reported in human salmonellosis (regardless of origin of the infection) are *S*. Entertidis (31.8%), *S*. Typhimurium (16.1%), *S*. Stanley (6.4%), *S*. Derby (6.0%) and *S*. Agona (2.5%). None of these serotypes were isolated from turtles in this study.

Phenotypic AMR was identified in eight (38.1%) and genotypic AMR in 11 (52.4%) of all 21 *Salmonella* isolates. The proportion of samples with AMR was relatively low compared to a similar study performed in wet markets in Shanghai where 100% (*n* = 82) of isolates showed resistance to at least one antimicrobial and 84.1% to at least three antimicrobials (17). A high level of AMR was also observed among pet reptiles in Spain, where 100% (*n* = 75) of *Salmonella* isolates were resistant to at least one of the 12 antimicrobials tested, and 72% were multidrug-resistant (36). Antimicrobial resistance genes for cephalosporin were detected in 19% (4/21) of isolates in this study. Furthermore, five quinolone ARGs were detected in seven different isolates. These findings are significant as cephalosporins and fluoroquinolones are the antimicrobials of choice to treat salmonellosis in humans and any *Salmonella* showing resistance to these drugs is a major concern for public health. Resistance to extended-spectrum cephalosporins and fluoroquinolones is particularly worrying since they represent the first-line antimicrobials to treat invasive salmonellosis in children and in adults respectively (39). Furthermore, the quinolone ARGs are significant in their ability to confer resistance by horizontal gene exchange (40).

In Hong Kong, 21% of *Salmonella* isolates collected between 2002 and 2004 from human cases were multidrug-resistant (41). The results from this and the current study are in stark contrast to findings from 1986 to 1996, where 99% of *S. enterica* serotype enteritidis strains isolated in Hong Kong were susceptible to 17 of the 19 antimicrobial agents tested (42), emphasizing the rapid emergence of AMR in *Salmonella enterica*. In the current study, resistance to chloramphenicol (33.3%, 7/21) and streptomycin (28.6%, 6/21) was the most common phenotypic AMR among the *Salmonella* isolates. The chloramphenicol and florfenicol resistance gene *floR* was one of the most common ARGs detected. Despite chloramphenicol being banned from aquaculture in mainland China since 2002 and streptomycin being a non-authorized antimicrobial drug, these two antimicrobials seem to be commonly used in aquaculture in mainland China (43). Furthermore, studies conducted in SS turtles for human consumption from mainland China in 2012 and 2016 showed that chloramphenicol residues could be detected in turtles' tissues. Thirteen antimicrobials have been authorized for use in aquaculture in mainland China: doxycycline, enrofloxacin, florfenicol, flumequine, neomycin, norfloxacin, oxolinic acid, sulfadiazine, sulfamethazine, sulfamethoxazole, sulfamonomethoxine, thiamphenicol and trimethoprim (43). Resistance against chloramphenicol and streptomycin in *Salmonella* isolates was also described in a study performed on pet turtles in South Korea, where 82.9% of *Salmonella* isolates were resistant against streptomycin (18). Similarly, these two antimicrobials are banned from use in aquaculture in South Korea. The authors hypothesized that these AMR patterns were due to the unregistered use of these drugs in pet turtles (18). However, this hypothesis could not be verified as the pet turtles were purchased from pet stores or online shops with no available information about their origin and breeding conditions. A similar hypothesis about the use of unauthorized antimicrobial drugs could be plausible for the current study.

Both *S*. Manhattan isolates were resistant against several antimicrobials. *Salmonella* Manhattan isolated from terrestrial wild iguanas (*Colonophus subcristatus*) from the Galapagos islands did not exhibit any resistance to antimicrobials (32). In the other two studies that identified *S*. Manhattan in captive iguanas (*Cyclura cychlura* and *Cyclura rileyi*) (33) and in northern water snakes (*Nerodia sipedon sipedon*) from Pennsylvania (34), no AMR testing was performed.

Similarly to the findings in turtles from wet markets in Shanghai (17), all *S*. Thompson isolates in this study were multidrug-resistant. While a phenotypic resistance against ciprofloxacin was detected, no ARGs against ciprofloxacin were found for this serotype. Resistance against ciprofloxacin is very problematic as it is the antimicrobial of choice to treat salmonellosis in humans. Ciprofloxacin-resistant *Salmonella* have increased in mainland China from 2.3% in 2006 to 5.9% in 2013 (44).

The *bla*_CMY−2_, which codes for the plasmid-mediated AmpC β-lactamase CMY-2 that hydrolyses third-generation cephalosporins (45) was detected in four isolates. Three of these isolates were phenotypically resistant to both third-generation cephalosporins tested by disk diffusion and the fourth had an intermediate phenotype. Because *bla*_CMY−2_ is encoded within a plasmid, it can be transmitted horizontally and spread among bacterial populations in animals and humans (46). The *bla*_TEM−1_ gene, which was found in the three S. Thomson isolates, codes for the TEM-1 β-lactamase. Mutation of this gene by only two single nucleotide polymorphisms (SNPs) can produce an ESBL capable of degrading third generation cephalosporins (47). The *bla*_TEM−1_ gene is amongst the most common ESBL genes found in Salmonella isolates in other studies (48, 49).

The *cfr* gene was detected in one *S*. IIIb 50:k:z isolate from a SS imported from Thailand. To the best of the author's knowledge, this is the first report of the *cfr* gene in *Salmonella*. This is an important finding as *cfr* confers resistance to five classes of antimicrobials, namely phenicols, lincosamides, oxazolidinones, pleuromutilins, and streptogramin A (50). Although oxazolidinones are not or only partially effective against Gram-negative bacteria due to their intrinsic resistance (51) the presence of the *cfr* gene in *Salmonella* could represent an additional reservoir for its potential horizontal spread to Gram-positive bacteria. The detection of *cfr* from *Salmonella* in turtles sold for consumption is particularly worrying as food is one of the main matrices responsible for transferring AMR determinants to humans. The *cfr* gene has also been identified in several species of staphylococcal bacteria (52), and in species within the genera *Bacillus, Enterococcus, Streptococcus, Macrococcus, Jeotgalicoccus, Proteus*, and *Escherichia* (53). It has also been detected in Methicillin-resistant *Staphylococcus aureus* (MRSA) strains from animals, humans (54) and food items (frozen dumpling) in mainland China (55). It was also identified in *Pasteurella multocida* isolated from poultry in mainland China (56).

In cases of salmonellosis that require antimicrobial treatment, the first-line of therapy is typically ciprofloxacin, azithromycin or the third-generation cephalosporin, ceftriaxone, although treatment options also include ampicillin and trimethoprim-sulfamethoxazole depending on the resistance profile (57). Worryingly, a phenotypic resistance and ARGs to all three commonly used first-line antimicrobial groups were detected in isolates from this study. *Salmonella* has been placed on the WHO high-priority list for the development of new antimicrobials because of the emergence of fluoroquinolone resistance (58).

An obvious sex bias was observed in our samples, as all SS were male and all RES and CSN were females. Sex determination in most turtles, including RES and CSN, is considered to be temperature-dependent, giving rise to males at lower temperatures and females at higher temperatures (i.e., over 30°C) (59, 60). In SS however, recent studies indicate that sex determination might have a genetic basis (61). Given the general lack of knowledge on turtle farming, it is difficult to make a hypothesis on the drivers of sex bias in the turtle market. Since sex and species are potential confounding variables, these results should be interpreted with care. The prevalence of *Salmonella* in several wild turtle species in the US was significantly higher in females than in males (62). This could potentially explain the differences observed in the current study, but it would require equal representation of both sexes in all three species.

The representativeness of the turtle population sampled needs to be interpreted with caution given the relatively small sample size and short sampling period. There is a lack of detailed information on turtle trade in Hong Kong SAR (i.e., turtle availability and volume in wet markets, species and sex distribution etc.) and data on *Salmonella* prevalence. Deciding on the right sampling strategy was challenging and the total number of samples taken from each retail location could not be adjusted accordingly. This project therefore used a pilot study approach. The results demonstrate a complex demographic structure (i.e., sex and species distribution) that might potentially persist even if a larger number of turtles is sampled.

Finally, the diversity of *Salmonella* serovars described here might be underestimated as only one isolation method and incubation temperature was used. However, the aim of the study was to generate some baseline data and to further characterize the isolated *Salmonella* strains using whole genome sequencing.

## Conclusion

This pilot study reports a high prevalence and serotype diversity of *Salmonella* among chelonians sold as food in Hong Kong wet markets, with the serotype *S*. Manhattan being—to the best of the author's knowledge—reported in chelonians for the first time. Resistance was detected against antimicrobials banned from aquaculture in mainland China and those recommended as first-line treatment for salmonellosis. The multidrug-resistance gene *cfr* is—to the best of the author's knowledge—reported for the first time in *Salmonella*. This is a worrying finding as it indicates an expansion of the *cfr* reservoir and the potential for horizontal spread to other bacteria. A systematic surveillance of *Salmonella* ideally from a representative sample of farmed turtles is essential to safeguard public health and for the timely detection of emerging threats.

## Data Availability Statement

The datasets presented in this study can be found in online repositories. The names of the repository/repositories and accession number(s) can be found in the article/supplementary material.

## Ethics Statement

The animal study was reviewed and approved by Animal Research Ethics Sub-Committee of City University of Hong Kong (Internal Ref: A-0592).

## Author Contributions

VC performed the necropsies and wrote the body of the manuscript. KL and HL performed culture and sensitivity testing. LW did the data analysis and assisted with manuscript writing. RP and CC assisted with study design and discussion writing. JH and MS carried out molecular analysis. RS assisted with laboratory data interpretation and discussion. IM developed the research idea and supervised the project. All authors contributed to the discussion and comments on the manuscript.

## Funding

This work was supported by the City University of Hong Kong start-up grant for new Faculty (project no. 9610449).

## Conflict of Interest

The authors declare that the research was conducted in the absence of any commercial or financial relationships that could be construed as a potential conflict of interest.

## Publisher's Note

All claims expressed in this article are solely those of the authors and do not necessarily represent those of their affiliated organizations, or those of the publisher, the editors and the reviewers. Any product that may be evaluated in this article, or claim that may be made by its manufacturer, is not guaranteed or endorsed by the publisher.
